# Physical Rehabilitation Programs for Bedridden Patients with Prolonged Immobility: A Scoping Review

**DOI:** 10.3390/ijerph19116420

**Published:** 2022-05-25

**Authors:** Remy Cardoso, Vitor Parola, Hugo Neves, Rafael A. Bernardes, Filipa Margarida Duque, Carla A. Mendes, Mónica Pimentel, Pedro Caetano, Fernando Petronilho, Carlos Albuquerque, Liliana B. Sousa, Cândida Malça, Rúben Durães, William Xavier, Pedro Parreira, João Apóstolo, Arménio Cruz

**Affiliations:** 1 Health Sciences Research Unit: Nursing (UICISA: E), Nursing School of Coimbra (ESEnfC), 3000 Coimbra, Portugal; hugoneves@esenfc.pt (H.N.); rafaelalvesbernardes@esenfc.pt (R.A.B.); margaridaduquee@esenfc.pt (F.M.D.); calexandracmendes@gmail.com (C.A.M.); monicapimentel@esenfc.pt (M.P.); fpetronilho@ese.uminho.pt (F.P.); cmalbuquerque@gmail.com (C.A.); baptliliana@esenfc.pt (L.B.S.); parreira@esenfc.pt (P.P.); apostolo@esenfc.pt (J.A.); 2 Portugal Centre for Evidence Based Practice: A JBI Centre of Excellence (PCEBP), 3000 Coimbra, Portugal; 3 Centro Hospitalar Universitário Cova da Beira (CHUCB), 6200 Covilhã, Portugal; pedrofscaetano@chcbeira.min-saude.pt; 4 School of Nursing, University of Minho, 4710 Braga, Portugal; 5 Health School, Polytechnic Institute of Viseu, 3500 Viseu, Portugal; 6 Mechanical Engineering Department, Institute of Engineering (ISEC), Polytechnic Institute of Coimbra (IPC), 3030 Coimbra, Portugal; candida@isec.pt; 7 Centre for Rapid and Sustainable Product Development (CDRSP), Polytechnic Institute of Leiria (IPL), 2430 Marinha Grande, Portugal; 8 ORTHOS SSI, Unipessoal LDA, 4809 Guimarães, Portugal; desenvolvimento.or5@orthosxxi.com; 9 WISEWARE, Lda., 3830 Ílhavo, Portugal; william@wisewaresolutions.com

**Keywords:** rehabilitation, rehabilitation exercise, programs, bedridden persons, review

## Abstract

Bedridden patients usually stay in bed for long periods, presenting several problems caused by immobility, leading to a long recovery process. Thus, identifying physical rehabilitation programs for bedridden patients with prolonged immobility requires urgent research. Therefore, this scoping review aimed to map existing physical rehabilitation programs for bedridden patients with prolonged immobility, the rehabilitation domains, the devices used, the parameters accessed, and the context in which these programs were performed. This scoping review, guided by the Joanna Briggs Institute’s (JBI) methodology and conducted in different databases (including grey literature), identified 475 articles, of which 27 were included in this review. The observed contexts included research institutes, hospitals, rehabilitation units, nursing homes, long-term units, and palliative care units. Most of the programs were directed to the musculoskeletal domain, predominantly toward the lower limbs. The devices used included lower limb mobilization, electrical stimulation, inclined planes, and cycle ergometers. Most of the evaluated parameters were musculoskeletal, cardiorespiratory, or vital signs. The variability of the programs, domains, devices and parameters found in this scoping review revealed no uniformity, a consequence of the personalization and individualization of care, which makes the development of a standard intervention program challenging.

## 1. Introduction

The developed societies currently face a severe demographic change: the world is aging at an unprecedented rate [1,2]. In 2050, the world population over 65 years old will near 1500 million people, about 22 percent of the world population [3]. With the increase in the average human lifespan, the number of older persons with mobility impairment, namely bedridden patients, is growing. In addition, bedridden patients caused by accidents and chronic progressive conditions are increasing yearly [4,5,6]. Recent studies have evidenced the economic impact on the institutions, highlighting that 25% of the resources might be used to care for bedridden patients [7,8] mainly due to the increased ventilator use and bed occupancy, which is why institutions are led to rethink recovery processes, namely seeking alternatives for acute care contexts.

Bedridden patients are usually kept in bed for long periods, slowly evidencing several problems caused by immobility [9]. Prolonged bed rest is the leading risk factor for the development of disuse syndrome, which causes significant systemic and organic pathological changes [10]. This is mainly due to the decompensation of the bedridden persons’ precarious physiological balance, after a significant reduction in their usual daily activities. Some manifestations are spatial-temporal disorientation, confusion, loss of automatic postural control, gait, and anatomic functional abnormalities. The latter involves all organs and apparatus and has psychological and metabolic repercussions [11,12].

The disuse syndrome, in the long term, increases the risk for the development of several conditions at a metabolic and systemic level. Some clinical entities to monitor and treat are joint contractures, sarcopenia, pressure sores, respiratory complications, and bone demineralization, which significantly decrease patients’ quality of life and delay the recovery process [13]. Many studies have shown that these complications are associated with increased morbidity and mortality [6,13], namely suggesting that 20 h of bed rest is sufficient to promote postural hypotension, and following 72 h, there is between 14 and 17% atrophy of muscle fibers [10].

Moreover, the impact of complications of immobility on patients’ overall well-being and functioning is a topic of growing interest in clinical research and practice [6,13,14]. Providing effective care, such as early rehabilitation, appears to hold promise in preventing disuse syndrome. This would involve early identification of the clinical signs associated with the development of immobilization syndrome and targeted interventions stressing mobilization, such as the prescription of simple exercises integrated into rehabilitation programs [15]. According to the available evidence, rehabilitation treatment can improve independence in patients with disuse syndrome; irrespective of the underlying cause [12,16]. Early mobilization is also an essential intervention among these patients. Although programs focusing on specific populations evidence positive effects [17], adequate, structured, and efficient programs seem to be lacking [18], considering that rehabilitation would be performed underlining personal goals and should be tailored to the main problem of the patient.

A shortage of those appropriately trained, a lack of demand, and a lack of human resources for rehabilitation constitute a severe strategic bottleneck for developing efficient institution and community-based services [19,20]. Therefore, providing human personnel with appropriate mechatronic devices or specialists with robots (integrating mechanics, electronics, sensors, intelligent control, actuators, and communication networks through integrated design) could reduce physicians’ physical and mental workloads [21]. Additionally, robots in rehabilitation therapies bring advantages over traditional treatments. They allow extensive practice in patients with substantial disabilities, reduce the effort required of therapists during the exercises, and provide a quantitative assessment of the patient’s motor function [22]. Ideally, these devices may allow the patient to do rehabilitation exercise training directly in bed without changing position, take up little space, and allow the patient to do rehabilitation in the hospital and at home [4].

Therefore, this scoping review’s main objective is to map the existing physical rehabilitation programs for bedridden patients with prolonged immobility. More specifically, the research questions are:

1. What are the physical rehabilitation programs for bedridden patients (e.g., neurological, orthopedic, and cardiorespiratory) with prolonged immobility?
2. What is the context where the physical rehabilitation programs are implemented (e.g., institutions, community care, and outpatient)?
3. What are the rehabilitation domains of the physical rehabilitation programs (motor, respiratory, and cardiorespiratory)?
4. What kind of devices are used for bedridden patients with prolonged immobility (e.g., elastics, weights, crankset, and EMS)?
5. What are the parameters assessed during the implementation of physical rehabilitation programs (e.g., muscle mass and oxygen saturation)?

## 2. Materials and Methods

This scoping review follows the Joanna Briggs Institute (JBI) [23,24,25] and complies with the Preferred Reporting Items for Systematic Reviews and Meta-Analysis extension for scoping reviews guidelines (PRISMA-ScR) [26]. A protocol for this review has been previously published (Parola et al. [27]).

### 2.1. Search Strategy

The databases searched included: MEDLINE (via PubMed), CINAHL complete (via EBSCOhost), Cochrane Central Register of Controlled Trials (via EBSCOhost), Cochrane Database of Systematic Reviews (via EBSCOhost), SciELO, Scopus, PEDro, SPORTDiscus with Full Text (via EBSCOhost), and Rehabilitation & Sports Medicine Source (via EBSCOhost). The search for unpublished studies included: DART-Europe and OpenGrey. The studies’ languages were limited to the ones mastered by the authors: English, Portuguese, and Spanish.

An initial limited search was undertaken on MEDLINE (via PubMed) and CINAHL Complete (via EBSCOhost) to identify articles on the topic. Consequently, the text words/expressions in the titles and abstracts of relevant articles and the index terms used to describe the articles were used to develop a complete search strategy across all the databases. Additionally, the reference list of all included papers was hand-searched for additional studies. The inclusion criteria of this scoping review were based on the “PCC” mnemonic: Population, Concept, and Context. Accordingly, this review considered studies: that included programs for bedridden patients with prolonged immobility (Population) that explored physical rehabilitation programs (Concept) conducted in any setting independently of the country of the study (Context). We also considered quantitative, qualitative, and mixed methods study designs for inclusion. Additionally, all types of systematic reviews were considered for inclusion independently of the publication date. The search strategy used in this scoping review can be seen in Table 1.

### 2.2. Data Extraction

All the records identified through database searching were retrieved and stored using Mendeley^®^ V1.19.8 software (Mendeley Ltd., Elsevier, The Netherlands), and any duplications were removed. All identified articles were accessed for relevance according to the title and abstract. The full text of the selected citations was assessed in detail against the inclusion criteria by two independent reviewers. The data were extracted from the articles and included in the review independently by two reviewers, using a data extraction table aligned with the objectives and research questions. The data extracted included: the author, year and country of the study, population characteristics, physical rehabilitation programs, accessed parameters, setting, and devices used. In the case of missing data, the study authors were contacted. Any disagreements regarding what data was relevant for extraction were resolved through discussion or with a third reviewer.

## 3. Results

### 3.1. Study Characteristics, Settings, and Sample

**Figure 1:**
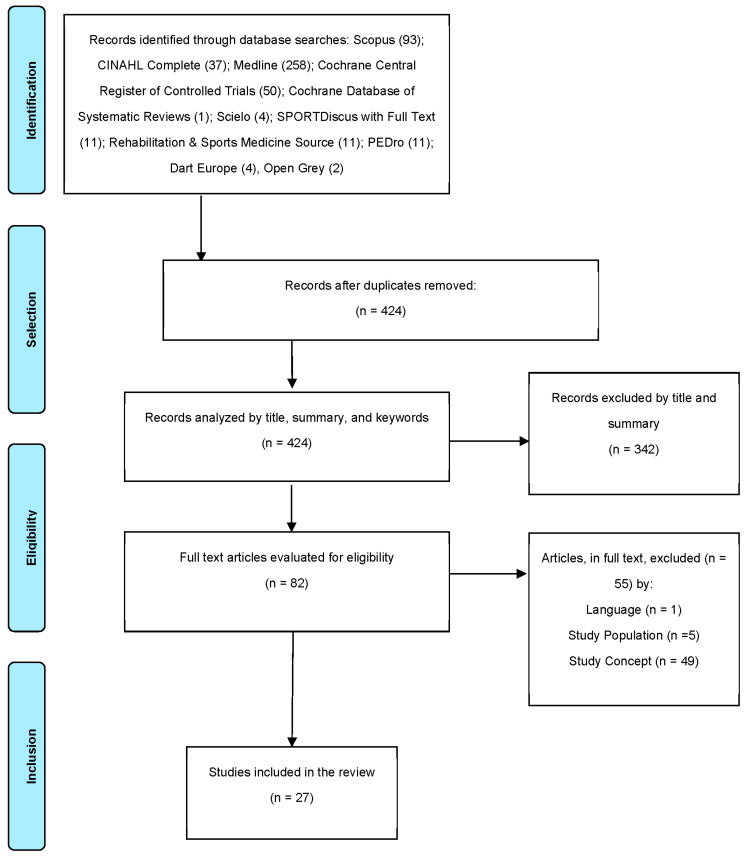
PRISMA flow diagram of the systematic review process.

A total of 424 potentially relevant studies (after duplicates were removed) were identified for study selection, and a total of 342 studies were excluded by evaluation of the title and abstract. The full-text versions of the remaining 82 articles were read, and 27 were found to fulfill the inclusion criteria (Figure 1). These 27 articles were published between 1999 and 2020 and, combined, represented intervention studies on 1476 subjects. The mean age reported in the included studies ranged from 21.6 to 85.4 years old, and most patients represented were male. The studies took place in Belgium (*n* = 1), Brazil (*n* = 1), China (*n* = 2), Croatia (*n* = 1), Denmark (*n* = 1), France (*n* = 2), Germany (*n* = 2), Italy (*n* = 3), Japan (*n* = 5), The Netherlands (*n* = 1), Poland (*n* = 1), Sweden (*n* = 1), Switzerland (*n* = 1), Turkey (*n* = 1), Taiwan (*n* = 2), and USA (*n* = 2) (Table 2).

### 3.2. Context

Most studies were performed in research institutes (*n* = 10) and hospital contexts (*n* = 10), followed on rehabilitation units (*n* = 3), welfare or nursing homes (*n* = 2), one long-term care facility (LTC) and one hospice. Studies performed in hospital context involved multiple conditions such as multi-trauma [54], hip fracture [45], sarcopenia [53], older patients with unspecified conditions [30,41,49], patients with COPD mechanical ventilated [42], or other mechanical ventilated patients [43], namely in the ICU [42,51]. Rehabilitation units involved patients with stroke [31], patients with multiple sclerosis [44], and one patient with head trauma caused by a car accident [38]. LTC facilities treated bedridden older stroke survivors [39]. In the welfare home, the programs were applied to bedridden patients with disuse syndrome [35] and in nursing homes for bedridden patients with multiple conditions but mainly stroke [50]. Finally, one study described a rehabilitation program in a hospice setting for palliative patients with fewer than three months’ life expectancy (mainly due to cancer diagnosis) [33].

### 3.3. Programs and Domains

Most rehabilitation programs were directed to the musculoskeletal domain (*n* = 14). The other seven were directed to the cardiorespiratory domain and six to mixed/other domains. Most of the programs included in this scoping were applied only to lower limbs [28,30,32,36,37,40,41], also including [34], which only focused on toe joints. On the other hand, two studies [33,39] worked on both upper and lower limbs, and three studies [29,31,38] worked on the upper and lower body. Regarding the programs in the cardiorespiratory domain, three studies [42,44,45] mainly focused on respiratory rehabilitation: one [46] was primarily cardiovascular, whereas three [43,47,48] worked on both the respiratory and cardiovascular systems. The other six [49,50,51,52,53,54] were classified as mixed or other domains because multiple domains were present, and the programs were not explicit or focused on other rehabilitation domains, such as [53], which combined nutrition, exercise, and pharmacotherapy. Given its variability from study to study, and that not all studies have this information, the aspects related to the duration of the study and sessions: frequency, intensity, and progressivity, are presented in Table 2.

### 3.4. Devices

More than half (*n* = 17; ~63%) of the above-described rehabilitation programs used rehabilitation devices. Of those devices, 11 were commercial devices, and the remaining 6 were prototypes. In the musculoskeletal programs, devices of different nature were used. Four studies [28,32,36,37] used devices for lower limb mobilization with different typologies (flywheel ergometer, leg press, and ankle or leg mobilizer), whereas one [40] used elastic bands, and [34] used a device for passive mobilization of toe joints. Devices for electrical stimulation were also widely used in the musculoskeletal [31,38,41], cardiorespiratory [42,46,48], and other/mixed domains [51]. Tilt tables for verticalization were also used in both musculoskeletal [31], cardiorespiratory [46], and mixed domains [49]. Two studies [31,46] used the commercial ERIGO tilt table, whereas the brand of this device used in one of the studies [49] was not specified. In the cardiorespiratory domain, the cycle ergometer was also common [43,48].

### 3.5. Clinical Parameters

A huge variety of parameters were accessed in the selected studies of this scoping to access the efficacy and safety of the above-described rehabilitation programs as the devices used in these programs. Most of the evaluated parameters were musculoskeletal, cardiorespiratory, or vital signs. In the musculoskeletal domain, the principal parameters accessed were muscular volume (using MRI) [28]. muscle strength [31,33,51] (mainly using the MRC scale), handgrip strength (using a dynamometer) [30], and force power (using a load cell) [28]. Still, in this domain, range of motion (ROM) [35,41], joint angular velocity [28], and angle measures [39] were also evaluated. Other techniques, such as dual-energy X-ray absorptiometry to measure bone mineral density [32] and EMG [28], were used.

In the cardiorespiratory domain, the main parameters evaluated for the cardiovascular system were electrocardiogram [43] and cardiac output using cardiac Doppler ultrasound [48], and lower limb blood flow [34,37]. For the respiratory system, the main parameters were tidal volume [43], forced vital capacity (FVC) [44,45], cough efficacy (pulmonary index) [44], peak cough flow (PCF) [45], and respiratory muscle strength using spirometry and maximal inspiratory and expiratory pressure (PImax and PEmax) measured with a manometer [44].

Vital signs [43] such as HR [31,42,46], RR [42,43,48], blood pressure: mean [31] systolic and diastolic blood pressure [46], SpO2 [31], and body temperature [52] were also frequently accessed. Two studies also evaluated neurological parameters such as brain activity using Electroencephalography (EEG) [36] and Near-Infrared Spectroscopy (NIRS) [36,48] and the memory performance using MRI and fMRI [47]. A variety of biomarkers were also accessed in some studies, such as urine and blood biomarkers of bone metabolism (e.g., 25-hydroxy- and 1,25-dihydroxy vitamin D, calcium, and osteocalcin) [32]; nutritional biomarkers (albumin) [30]; inflammatory biomarkers such as interleukins [42] and c-reactive protein [30,42]; and renal function biomarkers such as creatinine and cystatin [53]. Other clinical information such as the history of weight loss, triceps skinfold, dietary records [30], calf and arm circumferences [30], and BMI [30,44,53] was also recorded in some studies.

## 4. Discussion

This scoping review mapped the different rehabilitation programs for bedridden patients described in 27 primary studies elaborated between 1999 and 2020, specifically including the domains studied, devices used, parameters accessed, and the context in which these programs were performed.

### 4.1. Context

Regarding the context, we observed that a large number of studies were performed in research institutes with healthy controls; thus, at the time of publication of these studies, the respective programs were not yet implemented in a clinical context. On the other hand, this also demonstrates the importance of performing preclinical and clinical studies with healthy individuals [55,56] to access the rehabilitation program’s safety, since bedridden persons are often in a great state of fragility with a considerable variety of diseases and comorbidities [13,14]. A large number of studies were also performed in a hospital context. In this context, we observed that programs were applied to people with very different diseases (from hip fracture to stroke). On the other hand, other studies in the hospital context [30,41] and welfare/nursing homes [35,50] included patients (mostly older) with disuse syndrome, irrespective of the underlying cause [12,16]. Two studies [42,51] were specifically applied in the ICU context, demonstrating the importance of rehabilitation in intensive care to be performed as soon as possible [57,58]. Since rehabilitation is a continuous process [59], it can be started in the hospital but continued in rehabilitation centers and LTC, as observed in [31,38,39,44]. We also noticed a lack of studies on rehabilitation programs applied at home, possibly due to some barriers to implementing rehabilitation programs at home [60,61].

### 4.2. Domains

In this scoping review, we observed that most of the programs were directed to the musculoskeletal domain and, more specifically, the lower limbs. A lack of motor ability in the lower extremity affects walking ability balance and increases the risk of a fall [62]; on the other hand, it is the primary determinant of an independent and productive life and ADL [39,62]. Regarding the programs in the cardiorespiratory domain, two studies mainly focused on respiratory rehabilitation in ventilated persons [42,43]. This is especially important in this time of COVID-19 due to the number of persons that must be mechanically ventilated. Siddiq et al. [57] conducted a scoping review based on 40 recent publications demonstrating pulmonary rehabilitation’s importance. In this article, Siddiq et al. argued that survivors weaned from mechanical ventilation are at a higher risk of developing post-intensive care syndrome and that respiratory rehabilitation should be started at the earliest possible opportunity [57]. However, we must stress that persons admitted to ICU due to COVID-19 or other causes will also need musculoskeletal rehabilitation since people who stay in the ICU are also at risk of developing post-intensive care syndromes, which are defined as “physical, cognition, and mental impairments that occur during ICU stay, after ICU discharge or hospital discharge, as well as in the long-term follow up of ICU patients” [63]. Indeed, the programs classified as mixed domains in Table 2 demonstrate the need in certain cases of rehabilitation that comprise different domains.

### 4.3. Devices

Evidence shows us how devices can be an essential complement to the care provided to bedridden users [31,41,48,64]. In fact, in this scoping review, more than 60% of the included studies used devices as a compliment. Of those, 11 were commercial, and the remaining 6 were prototypes. Thus, although professionals already use commercial devices, an investment in the development of new devices adjusted to the population’s specific needs continues to be necessary. These devices are intended to fill gaps to which existing devices cannot yet respond [64]. In this scoping review, we also found that studies in the aerospatial scope [28,29] can be transported to the reality of clinical practice, even though their use was in a different scope. These studies focus on muscle and bone loss, which is a reality observed in long-duration missions in orbit and bedridden patients.

### 4.4. Parameters

This study observed a significant variability of parameters used to evaluate the implemented programs. The use of different parameters is often due to the study’s specific objectives, the contexts where they are implemented, the specificities of the population being studied, and the available resources. It is important to emphasize the adequacy of using the selected parameters concerning what is intended to be evaluated. However, the significant variability of the evaluated parameters may severely impair study comparison. This can pose a challenge for the development of, for example, systematic reviews of effectiveness, as there is no homogeneity between studies to carry out a meta-analysis [65]. The parameters most used in the different studies concern vital signs, namely the heart and the respiratory rates. Their use is essential for monitoring the safety of studies that focus on interventions for bedridden patients. Another observed aspect was the absence of specific information regarding muscular and osteoarticular risks, specifically in the control of joint stability during movement, a relevant aspect, especially when talking about complementary/robotic devices [66].

### 4.5. Limitations

In this scoping review, we subdivided the programs into musculoskeletal and cardiorespiratory domains. However, the line that separates them can be thin, because programs directed to the musculoskeletal domain can also benefit the cardiorespiratory domain and vice versa. Another limitation was that some studies did not present part of the information we intended to map, such as a complete characterization of the population, rehabilitation programs, or devices used, making it difficult to obtain all the information intended from the studies individually. Despite this limitation, we tried to extract as much information as possible from different studies to map all the available evidence. Another potential limitation of this scoping review was that only studies published in English, Portuguese, and Spanish were included. Articles published in other languages may potentially add information to the results of this review. Furthermore, since the objective of this scoping review was to map the physical rehabilitation programs for bedridden patients with prolonged immobility, no rating of the methodological quality was used. Although a critical appraisal of the included studies was not evaluated, since it was not relevant for the scoping review, some limitations were reported to provide valuable information to future research studies/systematic reviews.

## 5. Conclusions

To date, no previous scoping reviews addressing this purpose have been found. Therefore, this scoping review constitutes a valuable starting point for analyzing and systematizing the rehabilitation programs used for bedridden patients. Additionally, which devices were used, the implementation context and the parameters accessed were analyzed.

There is a great diversity of programs with different structures and variability in both devices and parameters to be evaluated. This review revealed no standardization of these components, making developing a standard intervention program challenging. This occurs since the programs and their components are adjusted to the specificities of the population under study, requiring individualization to meet the individual needs of specific patients. According to this evidence, rehabilitation treatment can improve independence in patients with immobilization syndrome, irrespective of the underlying cause, as described previously by Bocciogne et al. [49].

Mapping the evidence about physical rehabilitation programs for bedridden patients with prolonged immobility contributes to understanding this phenomenon, helping health professionals and stakeholders develop more adjusted programs and which parameters should be considered. Therefore, this mapping contributed to the identification of relevant issues to help advance evidence-based rehabilitation interventions, construct knowledge, identify gaps, and inform systematic reviews.

## Figures and Tables

**Table 1:** Search strategy.

**Medline (via PubMed)—searched on 8th July 2021: 258 results** ((((rehabilitation [MeSH Terms]) OR (rehabilitation [Title/Abstract])) OR (((((exercise*[Title/Abstract]) OR (Exercise Movement Techniques[MeSH Terms])) OR (Rehabilitation Exercise[MeSH Terms])) OR (Exercise[MeSH Terms])) AND ((bedridden[Title/Abstract]) OR (bedridden persons[MeSH Terms])) Filters: English, Portuguese, Spanish, MEDLINE
**CINAHL Complete—searched on 8th July 2021: 37 results** (TI rehab* OR AB rehab* OR TI exercise* OR AB exercise* OR ((TI physical OR AB physical) N1 (TI activit* OR AB activit*)) OR (MH “Therapeutic Exercise”) OR (MH “Physical Activity”) OR (MH “Exercise”) OR (MH “Rehabilitation”)) AND (TI bedridden OR AB bedridden OR (MH “Bedridden Persons”)) Limiters—Exclude MEDLINE records; Language: English, Portuguese, Spanish
**Cochrane Central Register of Controlled Trials—searched on 8th July 2021: 50 results** (TI rehab* OR AB rehab* OR TI exercise* OR AB exercise* OR ((TI physical OR AB physical) N1 (TI activit* OR AB activit*))) AND (TI bedridden OR AB bedridden)
**Cochrane Database of Systematic Reviews—searched on 8th July 2021: 1 result** (TI rehab* OR AB rehab* OR TI exercise* OR AB exercise* OR ((TI physical OR AB physical) N1 (TI activit* OR AB activit*))) AND (TI bedridden OR AB bedridden) **Scopus—searched on 8th July 2021: 93 results** ((TITLE-ABS (rehabilitation) OR TITLE-ABS (exercise*)) AND (TITLE-ABS (bedridden))) AND NOT ((PMID (1*) OR PMID (2*) OR PMID (3*) OR PMID (4*) OR PMID (5*) OR PMID (6*) OR PMID (7*) OR PMID (8*) OR PMID (9*))) AND (LIMIT-TO (LANGUAGE, “English”) OR LIMIT-TO (LANGUAGE, “Portuguese”) OR LIMIT-TO (LANGUAGE, “Spanish”))
**Scielo—searched on 8th July 2021: 4 results** (ti:(rehabilitation OR exercise*)) OR (ab:(rehabilitation OR exercise*)) AND (ti:(bedridden)) OR (ab:(bedridden))
**SPORTDiscus with Full Text—searched on 8th July 2021: 11 results** (ti:(rehabilitation OR exercise)) OR (ab:(rehabilitation OR exercise)) AND (ti:(bedridden)) OR (ab:(bedridden)) Filters: English, Portuguese, Spanish
**Rehabilitation & Sports Medicine Source- searched on 8th July 2021: 11 results** (ti:(rehabilitation OR exercise)) OR (ab:(rehabilitation OR exercise)) AND (ti:(bedridden)) OR (ab:(bedridden)) Filters: English, Portuguese, Spanish
**DART-Europe—searched on 27th November 2019: 4 results** (rehabilitation OR exercise*) AND bedridden Filters: English; Portuguese; Spanish;
**Open Grey- searched on 8th July 2021: 2 Results** bedridden
**PEDro—Physiotherapy Evidence Database—searched on 8th July 2021: 11 Results** bedridden exercise* = 10 OR bedridden activit* = 5 Filters: English; Portuguese; Spanish

**Table 2:** Articles including physical rehabilitation programs for bedridden patients with prolonged immobility.

Author, Year, Country	Population	Physical Rehabilitation Programs	Parameters	Context	Devices
Musculoskeletal Domain (*n* = 15)					
**Alkner et al.** [28] 2004, Sweden	Seventeen healthy subjects (26–41 years). Study subjects were divided into two groups: with (8) or without (9) resistance exercise	The training was performed in the 6° head-down tilt position. Study subjects performed four sets of 7 repetitions of supine squat and 14 repetitions of calf press every third day using a gravity-independent flywheel ergometer for 29 days; 2 min of rest was allowed between sets and 5 min between exercises	Quadriceps and triceps muscle volume using MRI (before and after bed rest). EMG, Peak force, power, work velocity, and minimum joint angle (for each repetition)	**Research institute**	Gravity-independent flywheel ergometer,
**Benjamin et al**. [29] 2009, USA	One healthy man (69 years)	The study subject used a newly designed bodysuit that applied precise loads to specific body parts	Artificial gravity force	**Research institute**	Bodysuit
**Blanc-Bisson et al.** [30] 2008, France	Seventy-six acute bedridden patients or with reduced mobility. (55F; 21M), 85.4 ± 6.6 years. Patients were divided into two groups: usual care and early physiotherapy program	Besides usual care, patients started on day 1 to 2: 10 repetitions of dynamic work against the foot of the bed (for triceps). When the subject was able to stand, exercises of plantar flexors and extensors were performed in the upright position. Extended leg, hip flexion at 45°/s alternatively for each leg; each repetition was maintained for 3 to 5 s, 10 repetitions with 10 s rest period between each (both legs). Knee flexed at 30°, moving pelvis to the left and the right, 10 repetitions (For the pelvis).	History of weight loss, weight, BMI, calf and arm circumference, triceps skinfold, day dietary records, serum albumin, and CRP. Katz index, handgrip strength, and change in ADL autonomy	**Hospital** (Acute-care geriatric medicine unit.)	Unspecified
**Calabro et al.** [31] 2015, Italy	Twenty bedridden patients due to ischemic stroke; 10 patients were randomly allocated in (G1) and 10 patients control group (G2)	Each G1—patient underwent 30 sessions of robotic verticalization procedures using the robotic tilt-table ERIGO. During the first 3 training sessions, patients were gradually verticalized from 10 to 30° over 15 min at a rate of 3° in 5 s. By session 5, verticalization was increased to 60° and reached 90° by session 10. During verticalization, each patient received a functional electrical stimulation treatment (30 mA of intensity). In G2, physiotherapy-assisted verticalization was performed through a simple tilt-table, with similar verticalization procedures to the G1.	The measure of the mean BP, HR, and SpO_2_. Lower limb’s paresis using the MRC scale, Patient’s postural control using the Postural Assessment Scale for Stroke patients	**Rehabilitation clinic** (neurorehabilitation)	ERIGO (Hocoma AG, Volketswil, Switzerland) Six-channel stimulator (Motionstim-8, Medel GmbH, Hamburg, Germany)
**English et al.** [32], 2014, USA	Forty healthy subjects (males) 34.9 ± 7 years. Study subjects were divided into six groups (8 per group according to concentric load)	Exercise testing and training were conducted using supine leg press and supine calf press exercise; Intensity (eccentric): 0% (concentric-only training), 33, 66, 100, or 138% of the concentric load. The program was performed 3 days per week over 12 weeks: 3 weeks of pre-testing, 8 weeks of training, and 1 week of post-testing,	Pre- and post-training whole body, lumbar spine, and hip bone mineral density. Whole-body lean tissue mass. Urine and blood markers of bone metabolism.	**Research institute**	Agaton Fitness System (Agaton Fitness AB, Boden, Sweden)
**Golčić et al.** [33] 2018, Croatia	Five hundred and thirty-six palliative patients with a life expectancy of fewer than 3 months (mainly cancer diagnosis), 71.77 ± 11.13 years. An approximate number of females (50.56%) and males.	The program consisted of active, actively assisted, and passive positioning exercises. Active exercises included the ability to move at least one of the arms or the legs against gravity. The exercises were considered actively assisted if the patients could start and perform the motion but were unable to complete the normal range of motion. Passive exercises consisted of stretching (5 to 10 repetitions) and performing ROM in at least all large joints of the extremities.	Manual-muscle test and performance score.	**Hospice**	Unspecified
**Ino et al.** [34] 2009, Japan	Ten healthy subjects (20 to 80 years)	Subjects’ toe joints were subjected to bending and stretching motions for 2 min, keeping the subjects in a supine position for 5 to 10 min	Lower limb blood flow	**Research institute**	Device for passive mobilization of toe joints
**Maimati et al.** [35] 2019, China	Eighty bedridden patients with disuse syndrome (50M; 30F), 69.25 ± 7.80 years were divided into experimental (40) and a control group (40)	Comprehensive rehabilitation nursing intervention was employed as follows: once a day, 50 min, 5 times a week and consisted of: Uyghur medicine; hand micro-vibration therapy, and training combined with education through a 20 min video once per week.	ROM of the hip joint, knee joint, and ankle joint.	**Welfare home**	Unspecified
**Pittaccio et al.** [36] 2013, Italy	Four healthy subjects (2 male; 2 female)	This program consisted of rest, active, passive, and assisted conditions for 5 min. Rest: the subject laid with the leg positioned on the leg rest of the mobilizer. Active: the subject performed a voluntary movement of the ankle, alternating 7 s of dorsiflexion hold to 30 s of relaxation. Passive and assisted conditions were performed with the Toe-Up! The device was set to produce cycles of 30° dorsiflexion (7 s) and relaxation towards plantar flexion in 30 s. In the passive condition: Toe-Up performed a continuous passive motion (CPM) to the subject’s ankle, whereas in the assisted condition, the subject was instructed to follow the CPM, collaborating actively in the dorsiflexion promoted by the device	Brain activity (EEG and NIRS in 4 different conditions: rest, active dorsiflexion of the ankle, passive mobilization of the ankle, and assisted motion of the same joint	**Research institute**	Toe-Up! An electro-mechanical mobilizer for the ankle joint
**Shimizu et al.** [37] 2017, Japan	Eight healthy subjects (5M; 3F); 21.6 ± 2.3 years, (19–25 years), Patients had no history (or risk factors) for deep vein thrombosis or lower limb operation	The participants performed 1-min leg exercise apparatus (LEX) exercises in three modes: (1) rapid single ankle motion (maximum active ankle dorsiflexion/plantarflexion at a rate of 60 cycles/min); (2) slow single ankle motion (maximum active ankle dorsiflexion/plantarflexion at a rate of 30 cycles/min); and (3) slow combined leg motion (active ankle dorsiflexion/plantarflexion and subtalar eversion/inversion at a rate of 30 cycles/min, with natural knee extension/flexion, hip/extension, and hip internal/external rotation).	Venous flow volume and velocity in the femoral vein at 1, 10, 20, and 30 min postexercise. These measurements were repeated three times for each participant,	**Research institute**	The LEX is a portable apparatus that enables patients to move their legs while supine.
**Talar et al.** [38] 2002, Poland	Twenty-eight-year-old male bedridden who suffered severe closed head injuries in an automobile accident and was in a comatose state for more than two months (GCS score of 5).	Physiotherapy was started with the patient still in comatose as follows: hydrotherapy, physical stimulation, including interference current (0.10 and 0.100 amps for 10 min, 6 times daily), low-power magnetic stimulation (15 min, 6 times daily), scanning laser (trunk and limbs, power 2J, 10 min, 6 times daily); manual massage of the trunk and limbs (30 min, 4–5 times per week). Kinesitherapy was initiated to restore locomotion after the patient awakened from the coma.	Clinical observation and family interviews. Wechsler Adult Intelligence, Vignos and Archibald scale. Rivermead Behavioural Memory, and FIM tests. Western Aphasia Battery; Frontal Behaviour Inventory and Boston Test of Praxis,	**Rehabilitation clinic**	Unspecified
**Tseng et al.** [39] 2007, Twain	Fifty-nine bedridden older stroke survivors: 17 in the usual care group, 21 in intervention group I, and 21 in intervention group II	Intervention group 1 involved a nurse supervising participants performing and completing the ROM protocol by themselves. Participants in intervention group 2 carried out the same ROM protocol with the nurse’s presence to help them physically achieve maximum ROM within or beyond their present ability; both intervention groups completed the ROM exercise protocol. This protocol was performed five times per joint, twice per day, and 6 days per week for 4 weeks, with each session lasting approximately 10–20 min.	17 joint angle measures in six joints (shoulder; elbow; wrist; hip; knee; dorsal ankle and plantar) and self-perception of pain using three ratings.	**Long-term care facilities**	Unspecified
**Vinstrup et al.** [40] 2017, Denmark	Twenty-two healthy subjects (15M/7F), 34.2 ± 14.7 years,	Elastic bands with a very low to very high resistance were attached to a standard-issued hospital bed. Total of 14 exercises: femoris muscle setting, prone knee extension, hip flexion with the leg bent, hip flexion with the leg straight, hip adduction, sideways hip abduction, prone hip abduction, supine knee flexion, hip thrust, dorsal flexion, plantar flexion, hip extension with the leg bent, and prone knee flexion performed with and without TheraBand Kinesiology Tape. The training session of 2.5 h and consisted of 3 repetitions of each exercise with 2 min of rest between exercises	Electromyographic signals were recorded from 13 lower extremity muscles. Borg CR-10 scale	**Research Centre**	Elastic bands (TheraBand CLX Consecutive Loops, TheraBand, Akron, OH, USA)
**Kataoka et al.** [41] 2017, Japan	Thirty bedridden disabled elderly patients	In addition to rehabilitation, the intervention mainly consisted of ROMex and sitting or standing; Belt electrode skeletal muscle electrical stimulation (B-SES) was applied on bilateral lower limbs 3 times per week for 3 months.	ROM of lower limbs at baseline and 1, 2, and 3 months after starting treatment, Muscle tone and pain	**Hospital**	B-SES
**Cardiorespiratory domain (*n* = 8)**					
**Akar et al.** [42] 2017, Turkey	Thirty COPD patients (15M) treated with Invasive mechanical ventilation were divided (blinded) into 3 groups (10 each): (1) active extremity exercise and NMES (2) NMES alone; and (3) active extremity ‘exercise alone	NMES was performed transcutaneously on the deltoid and quadriceps muscles using a four-channel neuromuscular electrical stimulator. The amplitude was switched between 20 mA and 25 mA (according to each patient). Symmetrical biphasic square waves with 6 s duration of contraction, 1.5 s of increase, and 0.75 s of decrease were applied. The wave frequency was 50 Hz. Patients were given a pulmonary rehabilitation program 5 days per week for 20 sessions.	Lower extremity and upper extremity muscle strength (scale of 5), mobilization duration, and weaning situation. Serum CRP, IL-6, IL-8, IL-10 and TNF-a, HR, RR.	**Hospital (ICU)**	Four-channel portable neuromuscular electrical stimulator, COMPEX device (MI Theta PRO, Switzerland)
**Chen et al.** [43] 2012, Twain	Twenty-seven prolonged mechanical ventilation patients	The cardiopulmonary exercise was performed on a cycle ergometer with a training intensity targeted at 60–80% of age-predicted maximal intermittent and short-term periods. Muscle-strengthening exercises included respiratory muscle and arm muscle strengthening exercises. Stretching exercises consisted of cervical, upper limb, and upper chest stretching. Respiratory muscle training was performed by putting a weight (0.5–2 kg sandbag) on the subject’s abdomen while he or she lay on the bed. The train lasted 30–40 min/session, 4–6 sessions/week for 10 sessions.	Vital signs and electrocardiogram. Physical functional status, pulmonary mechanics, ADL, BI, tidal volume, minute volume, respiratory muscle strength (maximal inspiratory pressure), and RR	**Hospital**	Ergometer (APT-5, Tzora, Kibbutz Tzora, Israel)
**Gosselink et al.** [44] 2000, Belgium	Twenty-eight bedridden (11) or wheelchair-bound (17) MS patients (13M) 58 ± 14 years were assigned to a training group (9) or a control group (*n* = 9)	The training group performed three series of 15 contractions against an expiratory resistance (60% maximum expiratory pressure (PEmax)) 2 times a day, whereas the control group performed breathing exercises to enhance maximal inspiration.	BMI, inspiratory and expiratory muscle strength (PImax and PEmax), FVC, neck flexion force, cough efficacy (Pulmonary Index); functional status (Extended Disability Status Scale.	**Rehabilitation center (for MS)**	Unspecified
**Guo et al.** [45] 2019, China	Eighty-four clinically stable patients with hip fractures who were aged above 65 years were randomly divided into either a yoga group (YG) (*n* = 42) or a control group (CG) (*n* = 42); 39 subjects in the YG (age, 74.10 ± 6.59 years) and 40 subjects in the CG (age, 75.10 ± 6.96 years) completed this study.	The “upper-body yoga” training was as follows: with closed eyes, the patient concentrated on breathing to inhale slowly and deeply through one’s nostrils, to raise his/her abdomen until the lung was fully expanded. Then, exhale completely through one’s mouth with a sound of “a~~” 10 times. Additionally, the patient rotated all joints of the upper limbs during a 1-min warm-up period. In the following phase, the patients inhaled deeply and raised one of their arms slowly to 180° from the front of the body, breathing quietly 3–5 times and exhaling completely with arms facing backward. Then, lean toward the left or right and breathe quietly 3–5 times before exhaling completely with arms facing backward. Then Inhale and exhale while simultaneously bending the elbows and rotating the shoulder joints as much as possible. In the final phase, the patient closes one’s eyes and breathes in and out quietly with his/her hands placed on the abdomen to relax and meditate for 3 min, followed by two quick and forceful breaths using the sound of “ha~”. The program was performed 2 times/day, 7 days/week	FVC/predicted value (FVC%), peak cough flow, BI, the incidence of pneumonia, rates of right skills, and inclination. Patients were tested in a 30° supine position on the day of admission (T1) after 7 days of training (T2) and 4 weeks after surgery (T3).	**Hospital**	Unspecified
**Tafreshi et al.** [46] 2017, Switzerland	Ten healthy participants	The study consisted of four different study protocols. (1) subjects were tilted to the maximum tilt angle of 71° and then to 40° with a 3 min supine period in between. In a second step, the same experiment was conducted at 60° instead of 71° (2 and 3) both protocols were conducted at = {20°, 40°, 60°} of tilt (three experiments per protocol) with or without FES, the FES frequency was set to 40 Hz. FES pulse was bipolar and biphasic with a width of 300 μs, and its amplitude could be varied between a minimum and a maximum (between 7 and 30 mA) (4). A 5-min synchronized stepping with minimum FES input was applied (uFES = 0, i.e., IMIN) followed by a 5-min interval of maximum FES input (uFES = 1, i.e., 0.8IMAX) and a 5-min period during which the amplitude was set back to the minimum current strength. The protocol was conducted at four different tilt angles = {0°, 20°, 40°, 60°} (to identify the effect of the change in FES amplitude during the stepping with FES on the cardiovascular variables)	HR, sBP, dBP	**Research institute**	ERIGO (Hocoma AG, Volketswil, Switzerland)
**Friedl-Werner et al.** [47], 2020, Germany	Twenty-three young, healthy men participants (29 ± 6 years) completed the study; 11 participants were randomly assigned to a high-intensity interval training (TRAIN)	The exercise training was performed in a supine position. Four different training sessions consisting of varying numbers of countermovement jumps and hops were designed and applied to TRAIN 5 to 6 training sessions per week for 60 days. The total training duration of one session did not exceed more than 17 min using an average training load between 80% and 90% of the body weight.	Memory performance and brain regions involved using MRI and functional magnetic resonance imaging (fMRI)	**Research institute**	Unspecified
**Medrinal, et al**. [48] 2016, France	Twenty participants	Exercises were divided into 10 min of passive ROM for the legs, 10 min of quadriceps electrical stimulation, 10 min of passive cycle-ergometry (MotoMed Letto II^®^), and 10 min of FES cycling (Reha-Move^®^) 20 rev/min for the last two exercises. For the exercises involving electrical stimulation, a rectangular, intermittent, bidirectional current with no ramp was used (length 300 μs, frequency 35 Hz). During FES cycling, electrical stimulation was synchronized with knee extension. A 30-min rest period was allowed between each intervention for the cardiorespiratory system to return to its baseline state	Cardiac output, pulmonary artery pressure, tricuspid annular plane systolic excursion (cardiac ultrasonography) Oxygenation of vastus lateralis muscle (NIRS). Expiratory volume and RR	**Hospital**	MotoMed Letto II^®^ RehaMove^®^, Hasomed, Germany
**Others/Mixed domains (*n* = 6)**					
**Boccignone et al.** [49] 1999, Italy	Fifty-four patients with disuse syndrome (28M, 26F), mean age 77 years)	Thirty minutes of rehabilitation treatments per session, 6 days per week. Treatment sessions included passive kinesitherapy, active kinesitherapy; early progression to sitting position; gradual progression to an upright position, exercises to restore gait,	FIM scale; Mini-Mental State Examination	**Geriatric hospital**	Tilt table
**Hirakawa et al**. [50] 2005, Japan	Fifty-three bedridden patients (>65 years) were divided into 2 groups: the home massage group (26) and the routine care group without massage (27)	Thirty-minute sessions of home massage rehabilitation therapy and kinesiotherapy by a massage practitioner 2 or 3 days a week for three consecutive months. Kinesitherapy: Sitting balanced exercises, sitting up exercises, Standing up exercises, Gait exercise, ROM exercises.	BI, Subjective Satisfaction and Refreshment Scale, Apathy Scale, and Self-rating Depression Scale at baseline and three months.	**3 home nursing stations, 13 visit care stations, and a one-day service center**	Unspecified
**Leite et al.** [51] 2018, Brazil	Sixty-seven subjects in mechanical ventilation were divided into 3 groups: control group (CG, *n* = 26), stimulation of quadriceps (Quadriceps group (QG), *n* = 24), and (stimulation of diaphragm (Diaphragm group (DG), *n* = 17).	The QG and DG patients received conventional physical therapy once a day, plus a daily electrical stimulation session from the first day of randomization until ICU discharge. For the NMES of the quadriceps, the following parameters were used: Aussie current, synchronized impulse at a frequency of 50 Hz, 1 s pulse increase period, 8 s “on” (muscle contraction) period, 1 s pulse decrease period, and 30 s “off” (disconnection) period. For the NMES of the diaphragm, the following parameters were used: Aussie current, synchronized impulse at a frequency of 30 Hz, 1 s pulse increase period, 1 s “on” (muscle contraction) period, 1 s pulse decrease period, and 20 s “off” (disconnection) period. Each session was performed for 45 min at intensities that produced visible contractions.	Length of hospitalization. Peripheral muscle strength (MRC). Respiratory muscle strength using a manovacuometer. BI and. Glasgow Coma Scale	**Hospital (ICU)**	Neurodyn MulticorrentesTM device (Ibramed, São Paulo, Brazil),
**Mendt et al.** [52] 2021, Germany	Sixteen healthy men (age: 30.5 ± 7.5 years) after 7 days and 49 days of Head-down tilt bed rest (HDBR). Five participants underwent HDBR only (CTR), 5 participants underwent HDBR and performed resistive exercises, and 6 performed HDBR and resistive exercises superimposed with vibrations (RVE).	Exercises were performed 3 times/week with a duration of 45 min. Exercise sessions were structured as follows: (1) short warm-up (bilateral leg press with 50% of pre-bed rest maximum voluntary contraction); (2) bilateral leg press (75–80% of maximum); (3) single-leg heel raises (about 1.3 times of their HDBR1 body weight); (4) double-leg heel raises (about 1.8 times of their HDBR1 body weight); and finally (5) back and forefoot raise (performing hip and lumbar spine extension against gravity with ankle dorsiflexion; a force 1.5 times body weight was applied at the shoulders). The RVE group additionally received vibration with frequencies between 16 and 26 Hz, depending on the exercise.	Core body temperature	**Research institute**	Tilt bed Galileo Space exercise device.
**Tatsumi et al**. [53] 2021, Japan	Seventy-year-old man bedridden man with sarcopenia developed as a postoperative complication	The patient was treated by initiating a 6- month-long Nutrition Support Team intervention that combined nutrition, exercise therapy, and pharmacotherapy. Priority was given to patient mobilization, the balance of energy intake and expenditure, prevention of complications associated with bed rest, and prevention of the progression of generalized deconditioning. Upper body muscle training was started 5 days a week for 20 min. Lower-limb muscle training was initiated to prevent the loss of skeletal muscle.	Weight, BMI, serum creatinine, eGFR creatinine, cystatin C, eGFR cystatin. Arm and arm muscle circumference	**Hospital**	Unspecified
**Bouman et al.** [54] 2008, The Netherlands	One hundred and thirty-two multi-trauma patients admitted to one of the Accident and Emergency Departments (A&E) participating hospitals are included.	Intervention group: Phase 1; There were 10 sessions per week of 30 min each. In addition, fitness, gymnastics, table tennis, swimming, bowling, hand bike, wheelchair training, and archery are given. There were 2–3 sessions per week for each treatment modality of 60 min each. Phase 2: new treatment aims were added by the physiotherapist. These might include a gradual individual weight-bearing scheme, coordination training, and functional training. There were 7 therapy sessions per week of 30 min. In addition, fitness, gymnastics, table tennis, swimming, rowing, cycling, and archery are given. This is offered in 2–4 sessions per week for each treatment modality of 60 min each.	Primary outcome measure: Generic quality of life: 36-item Short-Form Health Survey (SF-36); Functional health status: FIM)	**Hospital**	Unspecified

ADL—Activities of daily living; BI—Barthel Index; BMI—Body mass index; BP—Blood Pressure; B-SES—Belt electrode skeletal muscle electrical stimulation; CG—Control group; COPD—Chronic obstructive pulmonary disease; CPM—Continuous passive motion; CRP—C-reactive protein; dBP—Diastolic blood pressures; DG—Diaphragm group; EEG—Electroencephalography; EMG—Electromyography; F—female; FES—Functional electrical stimulation; FIM—Functional independence measure; fMRI—Functional magnetic resonance imaging; FVC—Forced vital capacity; G1—Group 1; G2—Group 2; HR—Heart rate; ICU—Intensive Care Unit; IL—Interleukin; LEX—Leg exercise apparatus; M—Male; MRC—Medical Research Council; MRI—Magnetic resonance imaging; MS—Multiple sclerosis; NIRS—Near Infra-Red Spectroscopy; NMES—Neuromuscular Electrical Stimulation; PEmax—maximum expiratory pressure; PImax—maximum inspiratory pressure; QG—Quadriceps group; ROM—Range of motion; RR—Risk Ratio; RVE—Resistive exercises superimposed with vibrations; sBP—Systolic blood pressures; TNF—Tumor Necrosis Factor; Y—years.
